# Development and evaluation of a Gal4-mediated LUC/GFP/GUS enhancer trap system in Arabidopsis

**DOI:** 10.1186/1471-2229-5-9

**Published:** 2005-06-07

**Authors:** Cawas B Engineer, Karen C Fitzsimmons, Jon J Schmuke, Stan B Dotson, Robert G Kranz

**Affiliations:** 1 Washington University, Department of Biology Campus Box 1137, 1 Brookings Drive St. Louis, MO 63130, USA; 2 Monsanto Company, St Louis, MO 63167, USA

## Abstract

**Background:**

Gal4 enhancer trap systems driving expression of LacZ and GFP reporters have been characterized and widely used in Drosophila. However, a Gal4 enhancer trap system in Arabidopsis has not been described in the primary literature. In Drosophila, the reporters possess a Gal4 upstream activation sequence (UAS) as five repeats (5XUAS) and lines that express Gal4 from tissue specific enhancers have also been used for the ectopic expression of any transgene (driven by a 5XUAS). While Gal4 transactivation has been demonstrated in Arabidopsis, wide use of a trap has not emerged in part because of the lack of detailed analysis, which is the purpose of the present study.

**Results:**

A key feature of this study is the use of luciferase (LUC) as the primary reporter and rsGFP-GUS as secondary reporters. Reporters driven by a 5XUAS are better suited in Arabidopsis than those containing a 1X or 2X UAS. A 5XUAS-LUC reporter is expressed at high levels in Arabidopsis lines transformed with Gal4 driven by the full, enhanced 35S promoter. In contrast, a minimum 35S (containing the TATA region) upstream of Gal4 acts as an enhancer trap system. Luciferase expression in trap lines of the T1, T2, and T3 generations are generally stable but by the T4 generation approximately 25% of the lines are significantly silenced. This silencing is reversed by growing plants on media containing 5-aza-2'-deoxycytidine. Quantitative multiplex RT-PCR on the Gal4 and LUC mRNA indicate that this silencing can occur at the level of Gal4 or LUC transcription. Production of a 10,000 event library and observations on screening, along with the potential for a Gal4 driver system in other plant species are discussed.

**Conclusion:**

The Gal4 trap system described here uses the 5XUAS-LUC and 5XUAS rsGFP-GUS as reporters and allows for *in planta *quantitative screening, including the rapid monitoring for silencing. We conclude that in about 75% of the cases silencing is at the level of transcription of the Gal4 transgene and is at an acceptable frequency to make the Gal4 trap system in Arabidopsis of value. This system will be useful for the isolation and comprehensive characterization of specific reporter and driver lines.

## Background

A number of different methods have been developed in *Arabidopsis thaliana *to discover and analyze promoters that are regulated by tissue specificity or environmental conditions [1-4]. These include the use of reporters GUS, GFP and more recently luciferase (LUC)[5,6] to detect "trapped" DNA elements that confer some type of regulation. Individual reporters and the exact type of trap system (e.g. gene fusion versus enhancer) each have their biases as to the type of expression element which is more frequently identified as well as their own advantages and disadvantages, which have been previously reviewed [7-9]. Of the available technical approaches, in the activator/upstream activating sequence (UAS) bipartite system, a transcriptional activator (factor) is used to trap endogenous enhancers. Transcription factor expression is detected using a reporter gene with the appropriate upstream UAS. This system has the advantage of using trapped lines to subsequently drive the ectopic expression of introduced transgenes. Trapping enhancer elements using the gene for the activator Gal4, which subsequently activates a reporter such as *lacZ *(possessing a Gal4 UAS), has been very successful in yeast and Drosophila [10]. The consequence for the fruitfly community has been the generation in the last ten years of well-defined Drosophila trap lines that show developmental or tissue specific expression of Gal4 [8,11]. These lines can then be used for expression studies with a reporter, typically *lacZ *or GFP, but also as driver lines to ectopically express any gene of interest that is placed downstream of the Gal4 UAS [12].

Enhancers are DNA regulatory elements that function over variable distance to alter the level of gene expression. An enhancer is generally not sufficient for gene expression and requires minimal promoter elements for transcription initiation. Enhancer elements provide much of the spatially, temporally and environmentally regulated gene expression in plants. Gal4 trap lines in Arabidopsis which allow rapid and quantitative analysis of gene expression levels will provide a valuable resource for understanding both tissue-specific and environmental regulation of gene expression. As a beginning, Haseloff has described a potential Gal4 trap system that uses a 5XUAS GFP (not red shifted, rs), whereby some Arabidopsis lines showed expression in the roots ([13], p 146–147 of ref. [14] and cited website [15]). The purpose of the present report is to describe the plasmids, silencing properties, and validation of Gal4-specific expression of our trap library in Arabidopsis. To develop an ideal Gal4 trap system, the reporter(s) should be quantitative, sensitive and stable over generations. Because of its long half-life, GFP is rarely used as a reporter for environmental gene regulation, and it is less feasible to perform quantitative expression studies with GFP *in planta*. We used luciferase as the primary reporter in this study, based on the short half life of luciferase and for its sensitivity and screening capabilities [5,16-18].

It was recently reported that in tobacco the Gal4 system is poorly expressed (i.e. silenced) because the high methylation status of cytosines (28–32%) impairs the binding of Gal4 to the UAS [19]. (Drosophila and yeast DNA is not methylated). The Gal4 binding site (UAS) typically contains GGCN11CCG and it is predicted from crystal structure studies that methylation would prevent binding [20], and methylation has been shown directly to inhibit binding [19]. The study in tobacco employed a 9 or 10X UAS repeat and the authors suggested that with less UAS repeats the affect due to methylation inhibition of Gal4 binding might be less, although this was not tested. *A. thaliana *possesses only 5% cytosine methylation [21] but the properties of a Gal4 trap system in this organism have not been documented in the primary literature. A previous study in Arabidopsis by Guyer et al used a 10XUAS-GUS reporter with a full 35S promoter driving Gal4 [22]. This study indicated that trans activation was feasible and no problems of silencing or methylation were noted. A question then remains whether Arabidopsis (and plants with methylation frequencies between those of Arabidopsis and tobacco) might be candidates to develop Gal4 trap systems for regulation or ectopic expression studies. The major goals of the present study were to 1) use a 5XUAS-LUC as reporter and determine if Gal4 driven by the full 35S promoter confers constitutive luciferase expression compared to a minimum 35S (thus acting as a trap); 2) determine whether a 5XUAS in Arabidopsis is optimal (compared to a 1X or 2X UAS); 3) determine stability of luciferase trap lines from seedling to seedling and over generations (i.e. silencing rates and basis); 4) produce a trap library with the potential for screening of three reporters (LUC, GUS, rsGFP), each activated by Gal4.

## Results and discussion

### T-DNA vectors for Gal4 studies using a 5XUAS-LUC reporter

**Figure 1:**
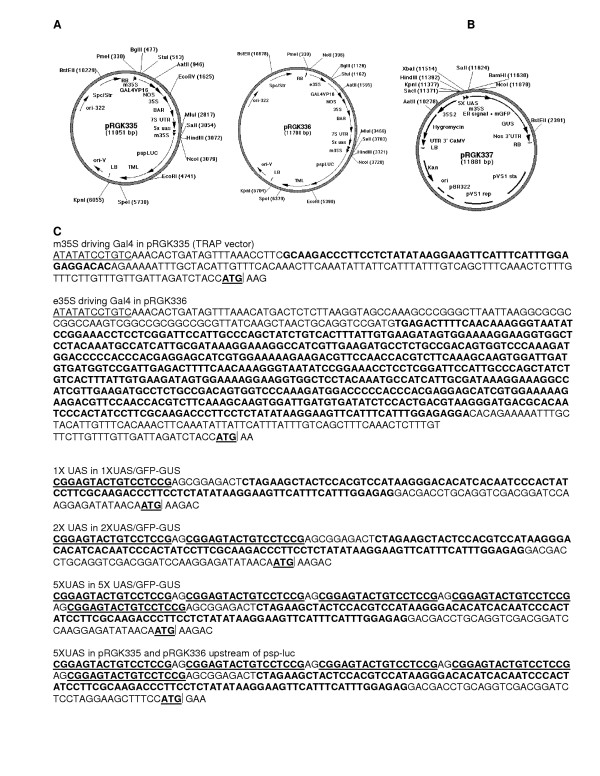
**Maps of plasmids and selected DNA sequences of constructs. **A, pRGK336 has enhanced 35S upstream of Gal4 while pRGK335 has minimal 35S upstream of Gal4. In both constructs, 5X UAS and m35S are upstream of the luciferase gene (psp-luc). B, There is no Gal4 in pRGK337and the 5XUAS and m35S are upstream of the GFP-GUS fusion protein. 2XUAS and 1XUAS versions are the same map but with the sequence changes noted in Fig 1C and the text. C, Selected DNA sequences of indicated regions. The right border is underlined and the TATA box is boxed; m35S and e35S are in bold. The ATG start site is bolded and underlined with an arrow for the indicated orfs. The UAS sequences are in bold and underlined showing the difference between 1X, 2X and 5X UAS.

The enhancer trap vectors constructed to examine the feasibility of a Gal4 activator/UAS system in Arabidopsis are diagrammed in Fig 1A and 1B and sequences upstream of the Gal4, LUC+ (pspLuc), and rsGFP-GUS genes are shown in Fig 1C. The first vector, pRGK336, has the full 35S promoter (e35S), including 35S enhancers, directly upstream of the Gal4 orf. (This orf is a hybrid of Gal4 DNA binding domain and the VP16 activation domain). The trap vector, pRGK335, only contains the minimum 35S TATA region. Both vectors possess the T-DNA right border directly upstream of the Gal4 promoter regions (Fig 1C). The *bar *gene encoding BASTA resistance was chosen for ease in selection of transgenic plants grown in soil. The LUC+ gene was inserted downstream of the minimum 35S and a 5XUAS (Fig 1C).

**Figure 2:**
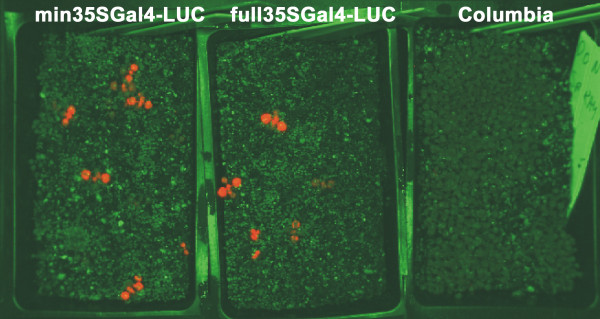
**Image of pRGK335, pRGK336 and untransformed plants. **Image of pRGK335 (left) and pRGK336 (middle) transformed plants and untransformed (right) in soil that have been sprayed with luciferin. Visible (artificially colored green) image has been overlaid with CCD captured luminescence (red).

**Figure 3:**
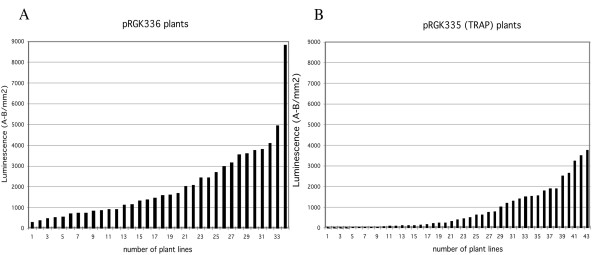
**Quantification of luminescence in individual plants grown in soil**. The indicated transformed seedlings were selected with BASTA, transplanted, sprayed with luciferin, and luciferase activity measured. Luciferase activity was normalized for background and area (A-B/mm2).

If the trap system functions in a Gal4-dependent manner, it is expected that the vector with the e35S promoter driving Gal4 expression (pRGK336) should show higher luciferase activities *in planta *than transformants with pRGK335 (the trap). Initially, approximately 300 seeds from transformed plants were sowed, sprayed with BASTA, and 10 days later sprayed with luciferin and imaged. Fig 2 shows an image of luciferase activity (light emission as shown in red) from these pots. A pot with control plants (i.e. Columbia) not sprayed with BASTA is shown on right. As expected, no Columbia plants exhibited luciferase activity. All BASTA-resistant plants transformed with pRGK336 showed luciferase expression and approximately half of the BASTA-resistant pRGK335 plants showed luciferase expression at the detection level shown. This validated that the luciferase gene can be expressed from both vectors. BASTA-resistant seedlings were transplanted (from BASTA selection media) and 14 days later luciferase activities were measured *in planta*. Relative levels in each seedling are shown in Fig 3, where zero delineates the Columbia control (i.e. no luciferase). In this experiment, for pRGK336 transformed plants, 32 out of 34 (94%) could be observed as expressors from digitized images of expression (as shown in Fig 2). The remaining two still had significant luciferase expression (200–300 relative units). A profile of the plants transformed with pRGK335 (trap vector) is shown in Fig 3. Approximately 50% were observed on digitized images and had activity above 200 relative units. The average luciferase activity for pRGK336 plants was 2054 units and for pRGK335 plants was 846 units. Thus, the plants appear to depend on the expected Gal4 expression levels for the luciferase expression. Taken together, both the higher percentage of seedlings which express luciferase and the higher level of expression observed with Gal4 expression from pRGK336 compared to pRGK335, suggests that this luciferase activity is dependent on the Gal4 expression.

**Figure 4:**
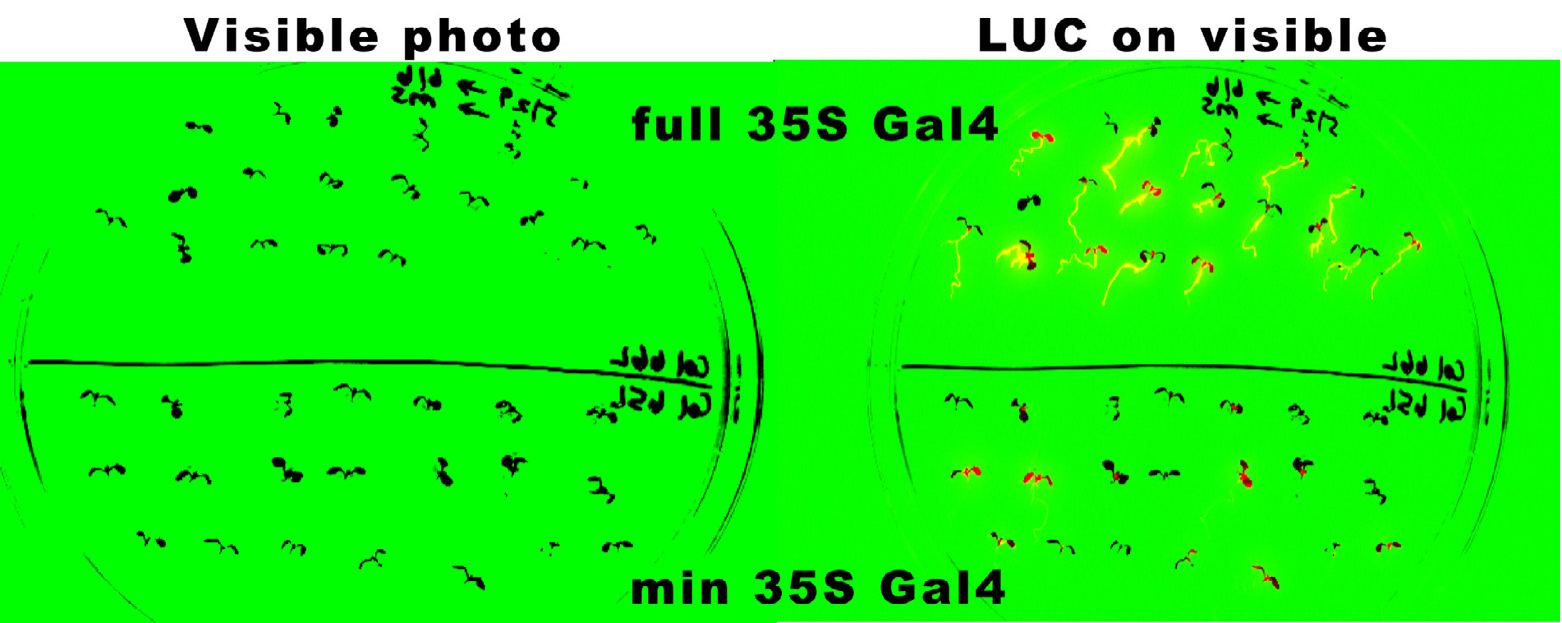
**Images of pRGK335 and pRGK336 plants. **Images of pRGK336 (top half of seedlings on each plate), pRGK335 (bottom half of seedlings on each plate) transformed plants on agar media with 50 uM luciferin. Left photo is visible CCD image, plants artificially colored black on a green background. Right photo has luciferase activity superimposed on visible – all yellow and red colors represent luciferase activity.

**Figure 5:**
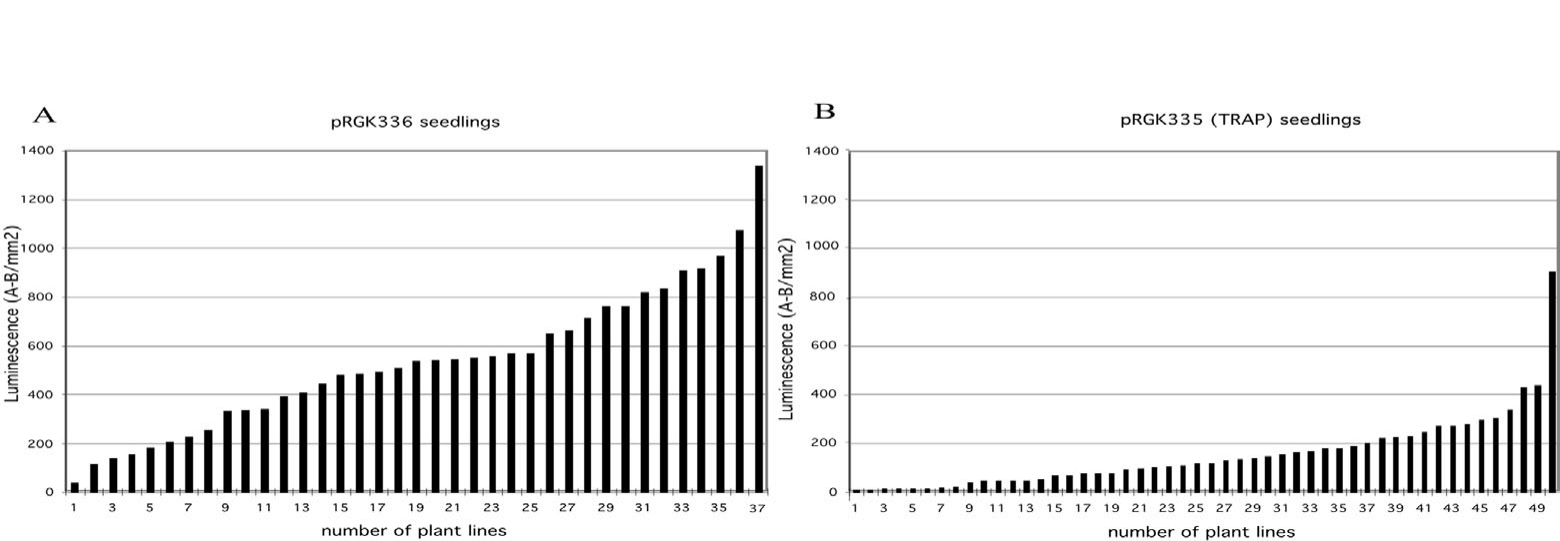
**Quantification of luminescence for seedlings grown on agar. **T1 seeds of the indicated transgenics were selected on BASTA, survivors transferred to media with 50 uM luciferin, and imaged. Luciferase activity was normalized for background and area (A-B/mm2).

Similar experiments were performed with seedlings at earlier stages of growth and agar media was used to examine luciferase activities in roots and aerial tissues. Eight day old seedlings were transferred after selection to agar plates containing MS medium with 50 uM luciferin and, after 24 hours, imaged for luciferase activity (Fig 4). Visible images of plants are shown in black on an artificial green background (Fig 4, Left) and the luciferase activities superimposed (Fig 4, Right), such that red or yellow indicate LUC expression. All but two seedlings with pRGK336 showed expression of luciferase in roots and cotyledons. Only 2 out of 21 pRGK335 plants showed expression in roots and 7 out of 21 in cotyledons. The variability in tissue specificity suggests that "trapped" enhancer elements drive Gal4 in the pRGK335 plants. Quantitation of luminescence in each seedling from a similar experiment is shown in Fig 5. Average luciferase activities in plants transformed with pRGK336 were 536 units and with pRGK335 were 151 units, again showing that expression is higher in plants that contain Gal4 driven by the e35S promoter.

**Figure 6:**
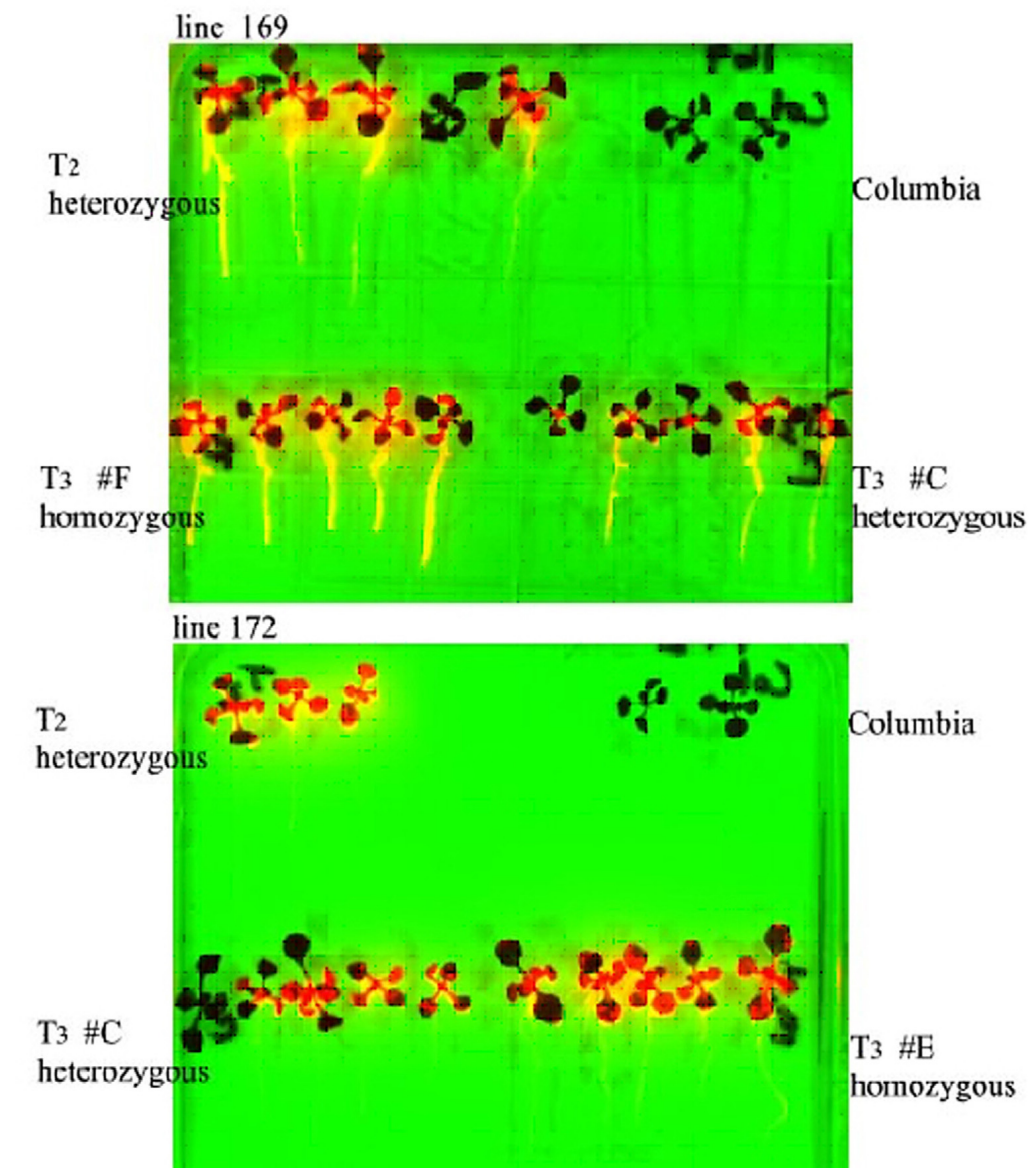
**Luciferase expression to determine variation and stability of homozygous and heterozygous seedlings. **Color indicators as shown in Fig 4 (red luciferase overlaid onto black visible: all yellow and red colors represent luciferase activity).

We determined inheritance ratios of the BASTA and LUC expression in T2 generations from the trap lines (with pRGK335). In a pool of T2 seeds, 55 out of 220 seedlings (25%) died on BASTA selection, suggesting a 3:1 overall inheritance of BASTA selection, as expected if the majority of transgenics are at a single locus. In a pool of T2 seeds under no selection, 52 out of 100 seedlings had luminescence when sprayed with 1 mM luciferin. Variations from one seedling to another in T3 generations of selected lines were also quantified. Fig 6 shows two examples of lines that were evaluated. T2 seedlings that were not under BASTA selection (called "T2 heterozygous" seedlings in Fig 6) were either heterozygous, homozygous or did not have the BASTA gene due to segregation. As expected, if BASTA resistance was lost, luciferase activity was also absent (e.g. see line 169, "T2 heterozygous" seedlings). Homozygous lines gave consistent expression from seedling to seedling with respect to luciferase levels and tissue specificity. This suggests that lines can be maintained as homozygotes and expected to express consistently within the T3 generation.

### Use of a 5XUASrsGFP-GUS secondary reporter and testing whether 5X, 2X, or 1X UAS repeats are better in Arabidopsis

Often, a secondary reporter (or phenotype) is useful for confirmation of a regulatory property or as an additional tool in understanding the expression of a gene. GFP and GUS reporters have their own advantages in use as reporters, so a system was developed that would potentially accommodate the use of all three reporters (LUC, GFP, GUS), each activated by Gal4. A GFP-GUS fusion protein (orf) was used for these studies (see below). Additionally, we wanted to determine whether a 1X, 2X, or 5X repeat would be better in Arabidopsis. A report by Johnstone and colleagues [23] has suggested that in yeast varying the numbers of UAS repeats can affect the "reporter" sensitivity with respect to levels of Gal4. As indicated above [19], it could also be advantageous to have fewer UAS repeats in plants because of the methylation silencing phenomenon.

**Figure 7:**
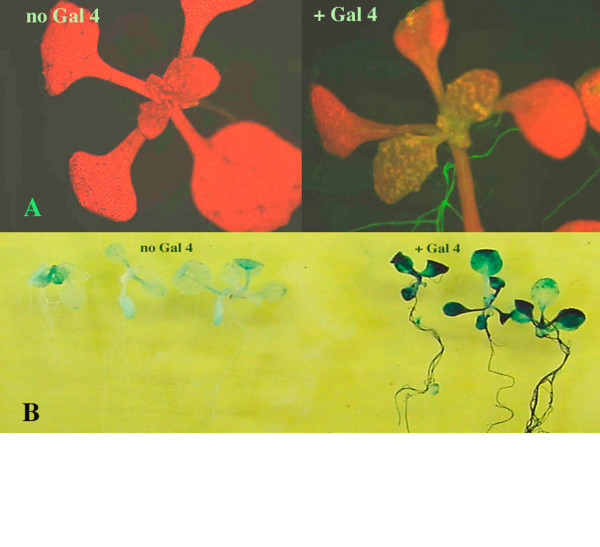
**GFP and GUS expression of the 5XUASrsGFP-GUS transgenic. **Loss of GFP-GUS expression when Gal4 is removed through segregation ("no GAL4") compared to line possessing GAL4 ("+GAL4"). A, GFP was viewed through a 500 nm GFP filter. B, Whole seedling GUS expression of indicated lines.

Arabidopsis was transformed with the three rsGFP-GUS vectors (1X, 2X, 5X UAS) diagrammed in Fig 1B. These are based on pCAMBIA1304, but the GFP was mutated to red-shifted GFP for higher quantum yield [24]. The serine at amino acid position 65 was changed to threonine by PCR based mutagenesis. The indicated UAS regions (with TATA from min35S) were cloned upstream (see sequences in Fig 1C). Columbia plants were transformed and selected with hygromycin. Next, we transformed the Gal4 vectors into various rsGFP-GUS transgenic lines to determine qualitatively which lines (1X, 2X, 5X UAS) expressed more GFP and/or GUS. Table 1 shows a qualitative assessment of their GUS activities in random transgenic plants possessing either a 1X, 2X, or 5X UAS and the Gal4 T-DNA. As expected, plants without T-DNA containing Gal4 did not express GFP, and the large majority did not express GUS or did so at low levels. The only combination that showed a majority of lines with high GUS activities were those with a 5XUAS rsGFP-GUS and the full e35S driving Gal4 (13 out of 22 lines). Some of these latter lines also exhibited GFP expression (see Fig 7 for examples of high GUS and GFP expressors). The 1X and 2X UAS lines showed weaker expression patterns and were therefore unsuitable for our future assays. We selected a single locus (5XUAS-rsGFP-GUS) line, named RGK1, that had high GUS and GFP which lost both activities when the Gal4 (BASTA-resistance) was segregated out in the next generation. A homozygous RGK1 line for this 5XUAS rsGFP-GUS reporter was chosen as the parent strain for producing a library using the pRGK335 trap vector (see below for library construction). The results with different numbers of UAS repeats indicate that in Arabidopsis the 5XUAS is optimal under the experimental methods described. Additionally, these qualitative data are consistent with the hypothesis that Gal4 levels dictate expression from the 5XUAS reporters.

### Silencing of the Gal4 system in Arabidopsis

To determine the frequency and properties of silencing in the Gal4 transgenics, we chose random lines of the pRGK336 and pRGK335 that exhibited significant luminescence in T2 seedlings and quantified expression in seedlings from T2, T3, and T4 generations. Initial studies on nine pRGK336 and nine pRGK335 lines indicated that no major drop in LUC expression from generations T2 to T3 occurred (not shown), with some lines decreasing and some increasing, but typically not by more than two to three fold.

A study of pRGK335 (trap) BASTA resistant lines was carried out on T2, T3, and T4 generations, where all three generations were grown on the same plates and assayed for luciferase activity (Table 2). Again, the changes from T2 to T3 were typically less than 3 fold. However, some lines were dramatically silenced when comparing the T2 to T4 generations: 5 out of 18 (28%) showed more than a 10 fold decrease in LUC expression with 4 out of 18 (22%) showing a greater than 100 fold decrease in expression in the T4 generation (relative to T2). Genetic analysis to determine the number of loci in these selected lines was based on T2 plant BASTA resistance/sensitivity ratios. Approximately 25% had an insert at a single locus, 50% had two loci and 25% had more than two loci. There was no correlation between silencing and number of inserts.

**Figure 8:**
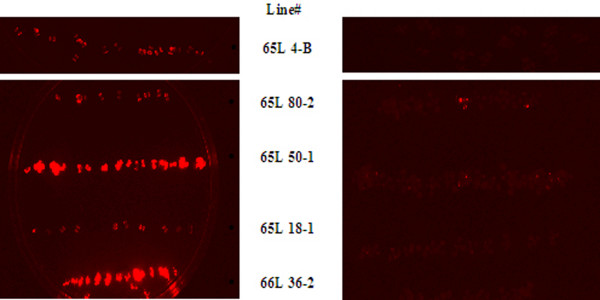
**Luciferase expression in silenced lines grown with and without AZA. **Luciferase expression of rows of the indicated "silenced" AZA (left) compared to media with no AZA (right). Red color indicates LUC activity. All seedlings were the same age. Line 66L 36-2 is a pRGK336 transgenic and all others are with pRGK335.

To determine whether this dramatic decrease in expression is due to methylation, seedlings from the T4 generation of each silenced line were grown in media with and without 5-aza-2'-deoxycytidine (AZA), an inhibitor of DNA methyltransferase. A silenced pRGK336 (e35S driving Gal4) line was also included in this study. In each of the lines, every seedling was recovered for LUC expression when grown on 7 ug/ml AZA (Fig 8). Quantitation of luciferase activities indicated that the highest recovered expression was with the pRGK336 line, and in general expression was directly proportional to the LUC activity observed in the T2 generation (not shown). This suggests that recovery is proportional to the levels of Gal4 expected in the cell and that silencing is due to methylation, possibly at the level of the 5XUAS (see next paragraph).

Silencing from T2 to T4 (or T3 to T4) generations could occur at the level of Gal4 or the 5XUAS-LUC transcription. To investigate this, mRNA was prepared from seedlings of various generations of selected lines immediately after imaging for luciferase activity. Multiplex Q-RT-PCR was used to determine the levels of Gal4 and LUC mRNA in these samples. Based on the constitutively expressed *Ubc-10 *gene, mRNA levels were determined. The levels of luciferase activity, Gal4 mRNA, and LUC mRNA are reported in Table 3. Control lines (169 and 213) that show only a minor decrease in luciferase activity also exhibited little change in Gal4 and LUC mRNA. Line 65L-4B is silenced mainly at the level of the 5XUAS LUC, while lines 50-1, 80-2 and 36 are silenced at the level of Gal4 and 5XUAS LUC transcription. Thus, both classes of silencing are observed (see Discussion).

### Library construction

With the single locus homozygous line RGK1 (referring to the 5XUASrsGFP-GUS described above) we have generated a library of approximately 10,000 events using the pRGK335 (trap) vector. Data reported in the previous section suggests that about 25% of the silenced lines will be silenced mainly at the 5XUAS LUC); a potential advantage to using the single locus line as recipient is the potential for less silencing in subsequent generations for the 5XUAS rsGFP-GUS (e.g. [25,26]. To ensure uniform representation of events in the library, 66 T1 plants were transplanted to a soil flat after BASTA selection. Each flat was harvested separately and seeds stored in an individual envelope. Equivalent weights of seeds from each individual tray was weighed out and combined with seed from 8 to 9 other trays. If plants had died, amount of seed was adjusted accordingly. Nineteen independent T2 seed pools of approximately 550 events each have been generated. Forty plants were studied further to compare luminescence, GUS and GFP levels and it was determined that the presence of GUS and GFP was related to high luciferase levels. For example, out of 21 plants showing greater than 1200 AU luminescence units, 13 showed GFP expression, whereas none of the plants with less than 1200 AU units exhibited detectable GFP. Screens for lines that exhibit tissue specificity and stable expression in subsequent generations are currently in progress. We have noticed no aberrant properties of transformed plants (e.g. stunted or chlorosis), suggesting that there are no deleterious effects due to the expression of LUC, rsGFP, GUS, Gal4, or *bar *in the library.

## Conclusion

A reliable, quantifiable enhancer trap system based on an activator/UAS approach in Arabidopsis was the principal aim of this research. The Gal4 activator was selected as the trans-activator for our system. While the Gal4 system in Drosophila has been quite useful, previous studies in tobacco revealed technical hurdles, such as methylation-induced silencing. In the tobacco study, a 9 or 10XUAS was used to drive GUS expression and Gal4 was expressed from a full 35S promoter [19]. Tobacco transgenics with this vector showed GUS expression in only 10 out of 60 lines (17%), quite different than the greater than 94% we observed here (with pRGK336). Additionally, specific tobacco transgenic expressor lines showed more than 20 fold variability in GUS expression from seedling to seedling, unlike with Arabidopsis shown here. Results with tobacco were shown to be erratic due to methylation of the UAS repeats, certainly a ramification of the high methylation frequency (32%) in that species [19]. Although we have not compared results using a 10XUAS repeat, a 5XUAS appears to be better suited for expression than a 1X or 2X UAS in Arabidopsis. We have proven that silencing by methylation in Arabidopsis will occur by the 4th generation in approximately 25% of the lines. On the other hand, 75% of lines retain expression through the T4 generation in our study (see below). Moreover, T3 seedlings appear to retain expression and for many applications this may be sufficient.

A general conclusion suggested from our study is that the higher the methylation status of an organism, the less useful a Gal4-type system will be, regardless of the number of UAS repeats employed. A very recent report [27] indicates "that Gal4/VP16-UAS elements provided a useful system for enhancer trap in rice". This is somewhat surprising because rice is approximately 19% methylated [21,28]. Wu et al. used a 6XUAS-GUS reporter and the GUS expression frequency in the initially transformed generation, called T0, was quite impressive (over 70% of transgenic lines) [27]. T1 generation plants had similar expression levels and tissue specificities (as T0), suggesting few effects due to methylation. No generations past the T1 were evaluated. The authors suggest that using a 6XUAS rather than 10XUAS, among a few other technical differences, might explain the lack of problems associated with methylation in rice when compared to that in tobacco [19]. Later generations in the rice system may begin to show silencing properties. Different species may also exhibit silencing effects due to methylation at different generations, and certainly the copy number of inserts will impact the silencing properties. Based on their results with tobacco, Moore and colleagues developed a different UAS bipartite system in which a modified lac repressor DNA-binding domain was combined with the Gal4 activation domain (called LhG4) [29]. This was at least proven technically feasible in tobacco and very recently, this has been applied to Arabidopsis to ectopically drive expression of a selected gene [30]. In this case, the driver lines expressed LhG4 from cloned promoter elements. A bipartite system that uses an ethanol-responsive AlcR activator with the *alcA *promoter for transactivation has recently been reported [31,32]. This system requires exogenous ethanol and also has been combined with tissue-specific promoters driving AlcR expression. This "ethanol switch" provides certain advantages, as described[31,32], and will have its own unique applications for driver line studies. We conclude from our results that random trapping using the more common Gal4:VP16 results in 25% silencing by the T4 generation but that much of this silencing may be at the level of the Gal4:VP16 transgene expression (Table 3). This suggests that any T-DNA delivered trap library (including LhG4 and AlcR) will result in some silencing in Arabidopsis and that methylation of the 5XUAS is not a major drawback.

## Methods

### Plasmid construction

The complete DNA sequences of the three plasmids shown in Figures 1A and 1B have been deposited in Gen Bank. Genbank accession numbers are AY739897 (pRGK335); AY739898 (pRGK336); AY739899 (pRGK337).

#### GFP-GUS vectors

The GFP-GUS fusion vectors are based on pCambia 1304, which has an engineered, fused GFP-GUS orf (Figure 1B). The GFP was changed to redshifted GFP to enhance detection [24]. A PCR product was made using the oligonucleotide primers rsGFP-F (5'CTGTTCCATGGCCAACACTTGTCACTACTTTC**A**CTTATGGTGTT) and rsGFP-reverse (5'AACGATCGGGG AAATTCGAG). The A in bold and underlined was changed from a T to an A to change a serine to a threonine. Both pCambia 1304 and the PCR product were digested with NcoI and BstEII and ligated, resulting in p1304-r-20.

A PCR product containing 5X UAS, m35S and an ER signal was made using the oligonucleotide primers UASCP7F1 (5'TAT**GGTACC**GATTACGCCAAGCTTGCATG) and 5XUAS/ER-REV (`TTGG**CCATGG**AACAGGTAGTTT) with pBinMGal4-VP16+UASmgfp5-ER (kindly provided by J. Haseloff, Cambridge University, see website [33] as the template. KpnI and NcoI sites are in bold. p1304-r-20 was digested with KpnI and NcoI to remove the LacZ alpha and the full 35S promoter. The PCR product was digested with KpnI and NcoI and ligated to p1304-r-20. The new plasmid with the 5XUAS is designated pRGK337.

To construct derivatives of rsGFP-GUS with 2XUAS and 1XUAS sequences, we took advantage of some natural, spontaneous deletions of the 5XUAS constructs. These contained a 1XUAS or 2XUAS with the sequences shown in Fig 1C. For a 2XUAS, we used the same right and left oligonucleotide primers as described for the pRGK337. Both the PCR product and p1304-r-20 were digested with KpnI and NcoI and ligated. For the 1XUAS version, a natural deletion within the Gal4 vectors described above was used as template. The oligonucleotide primers 5XUAS/ER-FWD (5`CAATA**GGTACC**TGAACGCGTCGGAGTACTG) and 5XUAS/ER-REV (5'TTGG**CCATGG**AACAGGTAGTTT) were used. NcoI and KpnI sites are in bold. Both the PCR product and p1304-r-20 were digested with KpnI and NcoI and ligated.

#### Gal4, LUC vectors

The pRGK336 and pRGK335 plasmids were constructed using pMON51850 as the core vector (containing the gene for bacterial spc/str-resistance), as diagrammed in Figure 1A. The construction of pRGK336 and pRGK335 were the same except for the insertion of the e35S and m35S respectively. These were constructed in the following steps to insert the indicated cassettes (the description begins at the right border as defined in Fig 1A): to insert the m35S and Gal4/VP16, a PCR product containing the m35S and Gal4VP16 from pBinMGal4-VP16+UASmgfp5-ER was used as template and the PmeI and BglII sites were added (sequences shown in Figure 1C); pRGK336 was constructed by replacing PmeI-BglII fragment with the full e35S promoter as a PmeI-BglII fragment (pMON23449); overlapping PCR from BglII to AvrII (in end of NOS 3'UTR) formed a cassette with the Gal4/VP16 orf (pBinMGal4-VP16+UASmgfp5-ER as template) and the NOS 3'UTR (pMON51850); GAL4PME-S (GATCGTTTAAACCTTCGCAAGACCCTTCCTC) GAL4NOS-S (GACGAGTACGGTGGGTAGCCCGATCGTTCAAACATTTGGC) GAL4NOS-R (GCCAAATGTTTGAACGATCGGGCTACCCACCGTACTCGTC) NOS-AVRII (GATCCCTCGGGATCTAGTAACATAGATGAC) an overlapping PCR product from the AvrII (in end of NOS 3'UTR) to MluI site formed a cassette with the BAR gene (pCGN9978) and 7S UTR (pMON51728); 35-AVRII (GATCCCTAQGGCTATCTGTCACTTCATC) BAR7S-S (CCTGCCCGTCACCGAGATTTGACCGTCCTTTGTCTTCAATTTTG) BAR7S-R (CAAAATTGAAGACAAAGGACGGTCAAATCTCGGTGACGGGCAGG) 7S-MLU-R (GATCACGCGTTCAATATTGTGGCAGGAC) for the 5XUAS-LUC-tml region, an MluI-BamHI PCR product (from pRGK337) contained the 5X UAS region; this intermediate plasmid was cut with Kpn1 and BamH1 and to insert the LUC gene, the luciferase vector, psp-luc+Vector, (Promega) was cut with BglII and KpnI and integrated. To leave only the LUC region, these were digested with EcoRI and KpnI and the TML UTR [34,35] was inserted as follows: a TML cassette was made using the oligonucleotide primers TML F1 (5' CGA**GAATTC**GGGAGGAAATTACACTGAGG) and TML R1 (5' CGGCAGGATATATTCAATTGTAAATTCC); the EcoRI site is in bold while the KpnI site is upstream of the primer [this cassette was derived from a plasmid that contained an overlapping PCR product formed from the TML (pCGN9978); UASTML-S (GATCACGCGTCGGAGTACTGTCCTCCG) GFPTML-S (CAACATGATGAGCTTTAACCCGGGTACCGAGCTC) GFPTML-R (GAGCTCGGTACCCGGGTTAAAGCTCATCATGTTTG) TMLSPE-R (GATCACTAGTTTTCAAATCCTTCAGATGG) and the 5Xtet region (pMON33643) TETSPE-S (GATCACTAGTCGTTAACTGCAGCTGAG) TETMFE-R (GATCCAATTGTAAATTCCTCTCCAAATGAAATGAAC) which contained the indicated KpnI site.] The TML cassette was digested with EcoRI and KpnI and ligated to form pRGK335.

### Plants and growth conditions in soil

*A. thaliana *ecotype Columbia-0 was used for all experiments. Plants in soil were grown in either long day or short day conditions. The long day growth chamber had 16 hours of light (175 uE) at 21°C. The short day chamber had 8 hours of light (175 uE) at 19°C. After seed set, plants were placed in a greenhouse with supplemental lighting to hasten drying. Soil medium was a mixture of Scotts Fafard germination mix, Scotts Rediearth and vermiculite #3, 1:1:1.4 respectively. Plants were fertilized once per week with Peters 15-16-17. Seedlings that were selected for BASTA resistance were sprayed with a 1:200 dilution of Finale concentrate at seven and fourteen days.

### Growth conditions of plants on agar

Plants were grown on MS salts and vitamins, 2% sucrose with pH 5.7 and .7% agar. Light regime was 16 hours of light (100 uE) at 22°C and 8 hours of darkness at 20°C. Agar plates were wrapped in Micropore surgical tape (3 M). Selection for plants on agar used hygromycin at 29 ug/ml or BASTA at 25 ug/ml. BASTA (glufosinate ammonium) was from Sigma. Nylon mesh used for seedling transfers is from Sefar America (catalogue # 06-300/34).

### Plant transformation

Plants were transformed via *Agrobacterium tumefaciens *using the floral dipping method [36]. *A. tumefaciens *ABIwas used for pRGK336 and pRGK335 transformations. *A. tumefaciens *GV3101 was used for all other transformations. Small scale cultures of 25 mL were grown overnight at 28°C in LB and selected antibiotics. Large scale cultures of 400 mL were inoculated with 1.5 mls of the overnight culture and grown to an OD600 of 1.3. Bacteria were spun down and resuspended to an OD600 of 0.6 in dipping solution consisting of 5% sucrose, 1 mg/ml BAP and 0.03% Silwet. Plants were dipped in the bacterial solution for 5 min, lightly drained, placed on their side and covered for 24 hours.

Plants were started on MS with 2% sucrose on 0.7% agar, with or without selection depending, on the plant background to be transformed. Seedlings were transplanted after 14 days to 2 1/2 inch pots, 4 plants per pot. Plants were moved to a short day growth chamber and covered with a dome for 1 day to aid acclimation. After 4 weeks, plants were moved to long day conditions; bolts were trimmed one time before dipping. Plants were dipped for transformation after 1 week in long day and dipped a second time 6 days later. Three weeks after the final transformation, plants were moved from the long day growth chamber to the greenhouse to hasten the drying process. Seeds were harvested when plants were completely dry.

### GFP expression

Seedlings were examined for GFP expression with a Zeiss Stemi SV11 microscope using a 500 nm GFP filter. Images were captured via AxioCam camera and software.

### GUS expression

Seedlings were stained for β-Glucuronidase activity as described previously [37]. Whole seedlings were immersed in a solution with 1.5 mM X-Gluc, vacuumed infiltrated 2 times for approximately 1 minute and incubated overnight at 37C in the dark. Tissues were destained via ethanol:acetic acid (3:1, v/v) before viewing. The X-Gluc solution consisted of 1.5 mM X-Gluc, 100 mM NaHPO_4 _buffer pH 7, 0.5 mM K_3 _[Fe(CN)_6_], 0.5 mM K_4 _[Fe(CN)_6_] and 10 mM EDTA.

### Luciferase imaging and quantitation

Firefly D-Luciferin, potassium salt (synthetic) was from Biosynth International. Luciferin was either sprayed on plants at 1 mM or incorporated into the media at 50 or 100 uM. For imaging, a Fuji LAS-1000 plus CCD Luminescent Image Analyzer from Fujifilm was used. Quantitation of luciferase activity (luminescence) in whole plants, roots, or other tissues used Science Lab 99-Image Gauge Ver. 3.4 software (Fujifilm).

#### Selection of T3 and T4 plants

6 plants from each T2 line were planted to soil and T3 seed were collected from these plants. This yielded 6 separate pools of T3 seed per line. These T3 seeds were assayed for BASTA resistance and homozygous lines were allowed to set seed (T4 seeds). These lines were used for the generation study in Table 2.

### RNA isolation, cDNA synthesis and multiplexed quantitative real-time PCR

Seeds were germinated on MS media and grown for 3 weeks with BASTA selection. Both generations being assayed for each plant line were grown on the same plate. Next, the plants were sprayed with 1 mM luciferin and imaged in the Fuji CCD camera and luciferase activities quantified. Tissue was harvested from whole plants and RNA extracted with the Qiagen RNeasy Plant kit according to the manufacturer's instructions (catalog# 74903). The RNA was treated with Invitrogen Amp grade DNase-I according to manufacturer's instructions (catalog# 18068-015). First-Strand cDNA synthesis was carried out on 2 μg of this RNA for each sample using the Invitrogen SuperScript III Reverse Transcriptase (catalog#18080). The cDNA was diluted 4-fold and used in subsequent Q-RT-PCR experiments. Light upon extension (LUX) primers for the Q-RT-PCR experiments were designed by Invitrogen Corporation's online LUX designer software and the sequences and fluorophore designations were as follows:

Gal4 Forward: CACTTGCCGCCTCAAGAAGCTCAAG [FAM] G

Gal4 Reverse: AGAGTAGCGACACTCCCAGTTGTT

Luciferase Forward: CACCGCTCTTCAATTCTTTATGCCGG [FAM] G

Luciferase Reverse: TGCGAAATGCCCATACTGTTG

UBC-10 Forward: CACTGCCTCGACATCTTGAAGGAGCAG [JOE] G

UBC-10 Reverse: GCTATCTCGGGCACCAAAGG

FAM = 6-carboxy-fluorescein

JOE = 6-carboxy-4', 5'-dichloro-2', 7'-dimethyoxy-fluoresein

Reactions were carried out with the Sigma Jumpstart *Taq *ReadyMix for quantitative PCR (catalog# D7440) according to manufacturer's instructions with the exception of using a final concentration of 4.5 mM MgCl_2 _for the Gal4 gene and 6.5 mM MgCl_2 _for the Luciferase gene. Cycling conditions included 94°C for 120 sec followed by 50 cycles of 94°C for 15 sec, 65°C for 30 sec and 72°C for 30 sec on the Cepheid Smart Cycler System. Every PCR reaction contained the primer pair for the Ubc-10 gene as an internal control in addition to either the Gal4 or luciferase primer pair. For each sample of cDNAs, reactions were carried out in triplicates. For each PCR tube, the ÄCt for the sample gene (either Gal4 or Luciferase) was calculated relative to the UBC-10 gene Ct and then the ÄCt.s were averaged for the triplicates. Fold differences in gene expression between samples were calculated by first determining the ÄÄCt values between samples and then using the formula: Fold Change = 2^(ΔΔCt)^.

## Distribution of material

All novel materials described in this study will be available for non-commercial research purposes. Contact RGK: Kranz@biology.wustl.edu

## Authors' contributions

CE was responsible for silencing studies, library screening methods and luciferase imaging optimization. KCF was responsible for construction of the three plasmids (as in Figure 1), luciferase imaging and library production and characterization. JS and SD were responsible for construction of plasmids on which some of those in Figure 1 are based and discussion of the studies. RGK is responsible for the design and execution of the study. RGK wrote the first few drafts while all authors participated in writing the paper.

## Figures and Tables

**Table 1:** GUS expression in 1X, 2X, and 5X UAS/GFP-GUS plants with different Gal4 backgrounds.

**No Gal4**			
	1X	2X	5X
# of Plants ^a^	6	11	8
Gus Expression:			
None	2	6	4
Low	4	4	4
High	0	1	0
**m35S/Gal4**			
	1X	2X	5X
# of Plants	14	15	17
Gus Expression:			
None	8	8	6
Low	3	4	6
High	3	3	5
**e35S/Gal4**			
	1X	2X	5X
# of Plants	26	15	22
Gus Expression:			
None	12	9	2
Low	7	4	7
High	7	2	13

^a ^T_1 _seedlings were intially selected on the appropriate chemicals (BASTA and/or hygromycin) and transplanted to soil. Two leaves from each plant were collected after two weeks in soil for Gus assay (as shown in Figure 7).

**Table 2:** Comparison of luminescence across generations for randomly selected trap lines.

**Line #**	**T2^a^**	**T3**	**T4**	**Fold Change**		**Fold Change**		**Fold Change**	
				**T2 to T3**		**T3 to T4**		**T2 to T4**	

172	6522	6145	5609	1.1	Same	1.1	Same	1.2	Same
216	2354	2485	2589	0.9	Same	1.0	Same	0.9	Same
									
25-1	16170	4990	51479	3.2	Decrease	10.3	Increase	3.2	Increase
169	12007	34529	24193	2.9	Increase	1.4	Decrease	2.0	Increase
173	4524	6452	6678	1.4	Increase	1.0	Same	1.5	Increase
									
212	5157	4150	3318	1.2	Same	1.3	Same	1.6	Decrease
27-4	9859	4283	5129	2.3	Decrease	1.2	Same	1.9	Decrease
47-1	6531	2680	3224	2.4	Decrease	1.2	Same	2.0	Decrease
14-1	3914	4110	1733	1.0	Same	2.4	Decrease	2.3	Decrease
213	2434	5286	895	2.2	Increase	5.9	Decrease	2.7	Decrease
214	3933	2171	1028	1.8	Decrease	2.1	Decrease	3.8	Decrease
211	8475	2423	1251	3.5	Decrease	1.9	Decrease	6.8	Decrease
77-2	1872	3668	200	2.0	Increase	18.0	Decrease	9.4	Decrease
215	11211	5995	479	1.9	Decrease	12.5	Decrease	23.4	Decrease
									
65L-4B	16022	2987	0	5.4	Decrease	3.0 × 10^3^	Decrease	1.6 × 10^4^	Decrease
18-1	1968	1163	0	1.7	Decrease	1.0 × 10^3^	Decrease	2.0 × 10^3^	Decrease
80-2	12378	6187	19	2.0	Decrease	3.0 × 10^2^	Decrease	6.0 × 10^2^	Decrease
50-1	16746	3867	19	4.3	Decrease	2.0 × 10^2^	Decrease	8.0 × 10^2^	Decrease

a. Luminescence was quantified for pRGK335 (trap) plant lines in multiple generations. Individual 14 day-old seedlings from each generation were grown on the same plates and whole plant luminescence quantified. Luminescence is expressed as Actual minus Background per mm^2^. 100 μM luciferin was included in the media. For the T2 lines, data is an average of 3 to 5 plants; only luminescent plants were included in the data (As described in the text, luminescence segregated/correlated with BASTA-resistance). For the T3 and T4 generations, all seedlings were imaged, as all were correlated with BASTA-resistance.

**Table 3:** Quantitative real-time PCR analysis of mRNA for Gal4 and LUC genes in selected lines^a^.

**Line#**	**Fold drop in LUC activity (generations used)^b^**	**Fold drop in gene expression**	
		
		**GAL4**	**LUC**
**169**	1.2 (T3/T4)	3	3.5
**213**	1.8(T3/T4)	2.3	3.3
**65L-4B**	42 (T2/T4)	1.6	147
**50-1**	33 (T2/T4)	59	56
**36^c^**	82 (T2/T4)	298	71
**80-2**	20 (T2/T4)	18	35

^a ^All numbers indicate a fold decrease in activity or expression as plant lines progress from the T2 (or T3) to the T4 generation.

^b ^Luciferase activity measured in BASTA-selected 14 day-old seedlings by spraying 1 mM luciferin.

^c ^This is a full 35S-driven Gal4 line as shown in Fig 8.
